# Clinical impact of near-infrared fluorescence imaging with indocyanine green on surgical treatment for hepatic masses in dogs

**DOI:** 10.1186/s12917-022-03467-2

**Published:** 2022-10-19

**Authors:** Naoki Sakurai, Kumiko Ishigaki, Kazuyuki Terai, Tatsuya Heishima, Kazuki Okada, Orie Yoshida, Yumiko Kagawa, Kazushi Asano

**Affiliations:** 1 Laboratory of Veterinary Surgery, Department of Veterinary Medicine, College of Bioresource Sciences, Nihon University, 1866, 252–0880 Kameino, Fujisawa, Kanagawa Japan; 2 North Labo, 35-8-2 Hondoori Shiraishi, 003-0027 Sapporo, Hokkaido Japan

**Keywords:** Dog, Fluorescence, Hepatic masses, Indocyanine green

## Abstract

**Background:**

Near-infrared fluorescence imaging using indocyanine green (ICG) is clinically applied to intraoperatively identify hepatic masses in humans. In addition, it is reported to be effective for assessing complete resection in human hepatocellular carcinoma (HCC). However, there is limited information on ICG fluorescence imaging for canine HCC, and its clinical usefulness is still unclear. Therefore, the purpose of this study was to evaluate the intraoperative identification and status of surgical margin for canine hepatic masses using near-infrared ICG fluorescence imaging. This clinical study included 104 dogs with hepatic masses. Between 12 and 24 h prior to surgery, ICG solution was injected intravenously at a dose of 0.5 mg/kg. The fluorescence intensity and pattern of each hepatic mass was investigated using an infrared camera before resection. After resection, the fluorescence intensity of the resection margin was also investigated. The resected masses were histopathologically diagnosed and compared using ICG fluorescence imaging.

**Results:**

One hundred and twenty-two masses obtained from 104 dogs included 76 HCCs, 16 hepatocellular adenomas, 12 focal nodular hyperplasias, and 18 other lesions. Of the 122 masses, 106 (94 partial, 9 whole, and 3 ring fluorescence patterns), 7, and 9 masses showed increased, the same, or decreased fluorescence compared to the normal liver tissue, respectively. The fluorescence intensity and pattern were not significantly related to the histopathological diagnosis. The sensitivity and specificity of the margin evaluation in the 47 dogs were 100% and 77.3%, respectively. The median survival times in cases of HCC with complete and incomplete resection were 914 and 254 days, respectively. The median survival time of patients with a complete resection was significantly longer than that of patients with a incomplete resection (*p* = 0.043).

**Conclusion:**

ICG fluorescence imaging has potential clinical value for the identification and margin evaluation of canine hepatic masses. Although it is difficult to use fluorescence imaging for the differential diagnosis of liver tumours, it may be useful for assessing complete resection in cases of hepatic masses demonstrating increased fluorescence in dogs, and complete resection of HCC could have a survival benefit.

## Background

Indocyanine green (ICG) is a cyanine fluorescent dye that emits light, peaking at approximately 835 nm when illuminated with near-infrared light (750–810 nm), and fluorescing at longer wavelengths of infrared light (835 nm) [1]. Intravenously administered ICG is selectively incorporated into hepatocytes and excreted into bile without being metabolized, entering the enterohepatic circulation, or being excreted by the kidneys. In human medicine, near-infrared (NIR) fluorescence imaging with ICG is applicable in an ever-expanding scope of clinical uses such as the identification of sentinel lymph nodes in various types of cancer (breast cancer, gastric cancer, lung cancer, esophageal cancer, etc.) [2–5] and graft patency for coronary artery disease [6]. Furthermore, ICG has been approved by the United States Food and Drug Administration for the determination of cardiac output, hepatic function, liver blood flow, and ophthalmic angiography [7]. With respect to veterinary medicine, ICG imaging has been used in several previous studies. These comprise: 12 hepatic nodules with hepatocellular carcinoma (HCC) in dogs [8], identification of the thoracic duct in dogs with chylothorax [9], sentinel lymph node evaluation in the oral cavities [10], intraoperative identification of parathyroid glands in [11], vascular visualization in caudal auricular flaps [12], and angiography for examining the normal ocular fundus [13]. However, due to the small number and sample sizes of such studies, much remains unknown.

Human HCC, one of the most commonly observed malignancies, develops as a result of hepatitis B and C viruses, alcoholic cirrhosis, and other diseases [14]. In human HCC, although ICG excretion is inhibited, portal uptake is preserved [15]. Furthermore, the excitation and emission of ICG is caused by infrared light; therefore, it is used to intraoperatively identify hepatic masses and has been shown to be effective for assessing complete resection [16–18].

Canine HCC is typically solitary and often develops from a single hepatic lobe [19–21]. The prognosis for dogs with massive HCC is good. In contrast, the prognosis for dogs with nodular and diffuse HCC is poor [20]. Surgery is the treatment of choice, and the outcomes are relatively favorable when complete surgical resection is possible [20, 22]. Regarding the relationship between complete margin resection and surgical outcome, whereas two studies concluded that complete resection was not associated with surgical outcomes [22, 23], another study found that complete resection extended the median survival time (MST) [24]. Primary malignant liver tumors, including cholangiocarcinomas, carcinoids, and sarcomas, are associated with poor prognosis due to recurrence and metastasis, even when the malignancies can be resected completely [25–29]. Thus, differential diagnosis of hepatic masses in canines and intraoperative assessment of their complete resection are clinically important.

Therefore, the aims of this study were to evaluate the intraoperative identification of hepatic masses via ICG fluorescence imaging in dogs, and to compare the status of their surgical margins as well as the findings of ICG fluorescence imaging.

## Results

The 104 dogs included in the present study had a median age of 11.8 years [range, 6.3–16.4 years] and median body weight of 7.3 kg [range, 2.5–44.0 kg]. Regarding sex, there were 45 sterilized females, 8 intact females, 38 castrated males, and 13 intact males. The breeds included were: miniature dachshunds (n = 16), Shih Tzu (n = 9), mixed-breeds (n = 9), Shiba Inus (n = 8), toy poodles (n = 8), French bulldogs (n = 6), Jack Russell terriers (n = 5), beagles (n = 4), Chihuahuas (n = 4), golden retrievers (n = 4), Yorkshire terriers (n = 3), miniature schnauzers (n = 3), border collies (n = 3), pomeranians (n = 2), Scottish terriers (n = 2), Shetland sheepdogs (n = 2), Labrador retrievers (n = 2), kishu (n = 1), English pointer (n = 1), Maltese (n = 1), Norfolk terrier (n = 1), Welsh terrier (n = 1), Siberian husky (n = 1), Airedale terrier (n = 1), Bernese mountain dog (n = 1), papillon (n = 1), standard poodle (n = 1), Samoyed (n = 1), American cocker spaniel (n = 1), Welsh corgi pembroke (n = 1), and German shepherd (n = 1). Table 1 summarizes the results of blood tests at the first evaluation. History of present illness included 3 cases of Cushing’s syndrome, 7 of hypothyroidism, 4 of epileptic seizures, 6 of mitral regurgitation, and 1 of chronic hepatitis and inflammatory bowel disease. Past medical history included 4 cases of mast cell tumor, 1 of anal sac adenocarcinoma, 2 of perianal adenoma, 2 of benign mixed breast tumor, 1 of granular cell tumor, and 1 each of nodular hyperplasia and lymphoid hyperplasia of the spleen.

**Table 1:** Preoperative blood tests

Variables	Unit		Median		Range		Reference	No. of cases	No. of cases above the reference (%)	No. of cases below the reference (%)
PCV	%		42		[18–57]		37–55	104	2.9	26.0
Hb	g/dL		14.4		[6.7–20.0]		12.0–18.0	104	0	23.1
RBC	10^6^/mL		6.57		[3.25–8.75]		5.50–8.50	104	3.8	21.2
WBC	/mL		8,600		[3,200–53,400]		6,000–17,000	104	6.7	10.6
Plt	10^3^/mL		540		[160–2,280]		200 − 500	104	57.7	1.0
TP	g/dL		7.1		[5.2–10.1]		5.2 − 8.2	104	1.9	0
Alb	g/dL		3.0		[2.2–3.9]		2.7–3.8	104	1.0	15.4
AST	U/L		43		[9–2,686]		17–44	104	47.1	4.8
ALT	U/L		207		[22–9,970]		10–100	104	81.7	0
ALP	U/L		899		[44–10,420]		23–212	104	79.8	0
GGT	U/L		13		[2–1,123]		0–7	104	78.8	0
TBil	mg/dL		0.1		[0.00–0.94]		0–0.9	92	1.1	0
BUN	mg/dL		16.0		[5.0–72.0]		7.0–21.0	104	26.0	4.8
Cr	mg/dL		0.7		[0.1–1.7]		0.5–1.8	104	0	20.2
Glu	mg/dL		100		[27–145]		77–125	104	7.8	12.6
NH_3_	mg/dL		17		[0–66]		16–75	41	32	31.7
Na	mmol/L		148		[140–154]		134–153	104	1.0	0
K	mmol/L		4.2		[3.3–5.6]		3.4–4.6	104	13.5	1.9
Cl	mmol/L		111		[102–118]		105–118	104	0	2.9
CRP	mg/dL		0.8		[0.0–20.0]		0–1.00	103	40.8	0
APTT	sec		13.4		[10.5–26.5]		9.5–26.5	101	17.8	1.0
PT	sec		6.9		[4.4–8.4]		6.0–8.0	101	4.0	21
Fib	mg/dL		296.9		[92.7–800.0]		86.0–375.0	101	26.7	0
AT	%		159		[88–229]		102–156	101	53.5	4.0
D-dimer	mg/dL		0.9		[0.0–118.2]		0–2.0	77	33.8	0

PCV, packed cell volume; Hb, hemoglobin; RBC, red blood cell count; WBC, white blood cell count; Plt, platelet count; TP, total protein; Alb, albumin; AST, aspartate aminotransferase; ALT, alanine aminotransferase; ALP, alkaline phosphatase; GGT, gamma- glutamyl transferase; TBil, total bilirubin; BUN, blood urea nitrogen; Cr, creatinine; Glu, glucose; NH_3_, ammonia; Na, sodium; K, potassium; Cl, chloride; CRP, C-reactive protein; APTT, activated partial thromboplastin time; PT, prothrombin time; Fib, fibrinogen; AT, anti-thrombin activity

Operative procedures consisted of left divisional hepatic lobectomy in 20 dogs, central divisional hepatic lobectomy in 7 dogs, right divisional hepatic lobectomy in 25 dogs, left and central divisional hepatic lobectomy in 1 dog, and central and right divisional hepatic lobectomy in 2 dogs. Other procedures performed are summarized in Table 2.

**Table 2:** Operative procedures in 104 dogs

Operative procedures			Number of cases
Left divisional hepatic lobectomy			20
Central divisional hepatic lobectomy			7
Central divisional hepatic lobectomy	+	Left lateral hepatic lobectomy	1
Central divisional hepatic lobectomy	+	Left medial hepatic lobectomy	1
Central divisional hepatic lobectomy	+	Right lateral hepatic lobectomy	1
Right divisional hepatic lobectomy			25
Right divisional hepatic lobectomy	+	Left lateral hepatic lobectomy	1
Left and central divisional hepatic lobectomy			1
Central and right divisional hepatic lobectomy			2
Left lateral hepatic lobectomy			22
Left lateral hepatic lobectomy	+	Left medial hepatic lobectomy	1
Left lateral hepatic lobectomy	+	Quadrate hepatic lobectomy	1
Left medial hepatic lobectomy			4
Left medial hepatic lobectomy	+	Caudate process resection	1
Quadrate hepatic lobectomy	+	Right medial hepatic lobectomy	1
Right medial hepatic lobectomy			3
Right lateral hepatic lobectomy			2
Right lateral hepatic lobectomy	+	Left medial hepatic lobectomy	1
right lateral hepatic lobectomy	+	Papillary process hepatic lobectomy	1
Papillary process hepatic lobectomy			4
Papillary process hepatic lobectomy	+	Left lateral and left medial hepatic lobectomy	1
Caudate process hepatic lobectomy			3

A total of 122 nodules were resected from 104 dogs with hepatic masses. Histopathologically, these nodules comprised 76 nodules with HCC (well-differentiated in 52 nodules, poorly differentiated in 24 nodules); 16 nodules with hepatocellular adenoma; 12 nodules with nodular hyperplasia; 5 nodules with primary sarcoma including 2 undifferentiated sarcoma, 2 hemangiosarcoma, and 1 liposarcoma; 4 nodules with hepatocholangiocarcinoma; 2 nodules each with liver abscess, bile duct adenoma, and metastatic neoplasia; and 1 nodule each with choledochal cyst, cholangiocarcinoma, and neuroendocrine tumor.

The fluorescence intensities and patterns are summarized in Table 3. Of the 122 nodules, 106 showed fluorescence intensity 1, which is increased fluorescence, 7 showed fluorescence intensity 0, which is no change in fluorescence; and 9 showed fluorescence intensity − 1, which is decreased fluorescence.

**Table 3:** Fluorescence intensity and Fluorescence pattern in 122 nodules

Microscopic diagnosis	Number of nodules	Fluorescence intensity			Fluorescence pattern		
		1	0	-1	Partial	Whole	Ring
		(n = 106)	(n = 7)	(n = 9)	(n = 94)	(n = 9)	(n = 3)
Well-differentiated HCC	52	47	2	3	42	5	0
Poorly differentiated HCC	24	22	0	2	21	1	0
Hepatocellular adenoma	16	13	1	2	13	0	0
Nodular hyperplasia	12	11	1	0	10	1	0
Primary sarcoma	5	5	0	0	3	1	1
Hepatocholangiocarcinoma	4	4	0	0	4	0	0
Liver abscess	2	1	0	1	0	0	1
Cholangiocarcinoma	1	0	0	1	0	0	0
Bile duct adenoma	2	1	1	0	0	1	0
Neuroendocrine tumor	1	1	0	0	1	0	0
Metastatic neoplasma	2	0	2	0	0	0	0
Choledochal cyst	1	1	0	0	0	0	1

Partial: partial fluorescence pattern, Whole: whole fluorescence pattern, Ring: ring fluorescence pattern, HCC: hepatocellular carcinoma

*Fluorescence patterns were evaluated for those with a fluorescence intensity of 1.

Fluorescence patterns were assessed for the 106 nodules that were fluorescent. A partial fluorescence pattern was observed for 94 nodules, among which 42 nodules had well-differentiated HCC, 21 had undifferentiated HCC, 13 had hepatocellular adenoma, 10 had nodular hyperplasia, 4 had hepatocholangiocarcinoma, 3 had primary sarcoma, and 1 had a neuroendocrine tumor. A whole fluorescence pattern was observed for 9 nodules, which comprised 5 nodules with well-differentiated HCC and one each with undifferentiated HCC, nodular hyperplasia, bile duct adenoma, and primary sarcoma. A ring fluorescence pattern was observed in 1 nodule each with a choledochal cyst, liver abscess, and primary sarcoma. Comparisons of histopathological findings and fluorescence pattern did not reveal any association between the two.

The fluorescence of the resection margin was assessed and compared among 47 cases. It was difficult to thoroughly evaluate the resection margins in some cases due to heavy intraoperative bleeding. In such cases, it was necessary to quickly finish the surgery to reduce hemorrhage, and fluorescence imaging was not performed. Therefore, assessment of fluorescence of the resection margin was achieved in 47 cases. All 3 cases of incomplete resection demonstrated fluorescence of the resection margin. Thirty-four out of 44 cases with complete resection did not demonstrate fluorescence of the resection margin, but the other 10 did. Regarding the diagnostic accuracy of complete resection according to ICG fluorescence imaging, we calculated a sensitivity of 100% and specificity of 77.2%. Thirty cases of HCC among the abovementioned 47 cases were similarly compared; two cases of incomplete resections both demonstrated fluorescence of the resection margin. Twenty-three of the 28 cases of complete resections did not demonstrate fluorescence of the resection margin, whereas the other 5 did. Regarding the diagnostic accuracy of complete resection according to ICG fluorescence imaging in cases of HCC, its sensitivity and specificity were 100% and 82.1%, respectively.

**Fig. 1:**
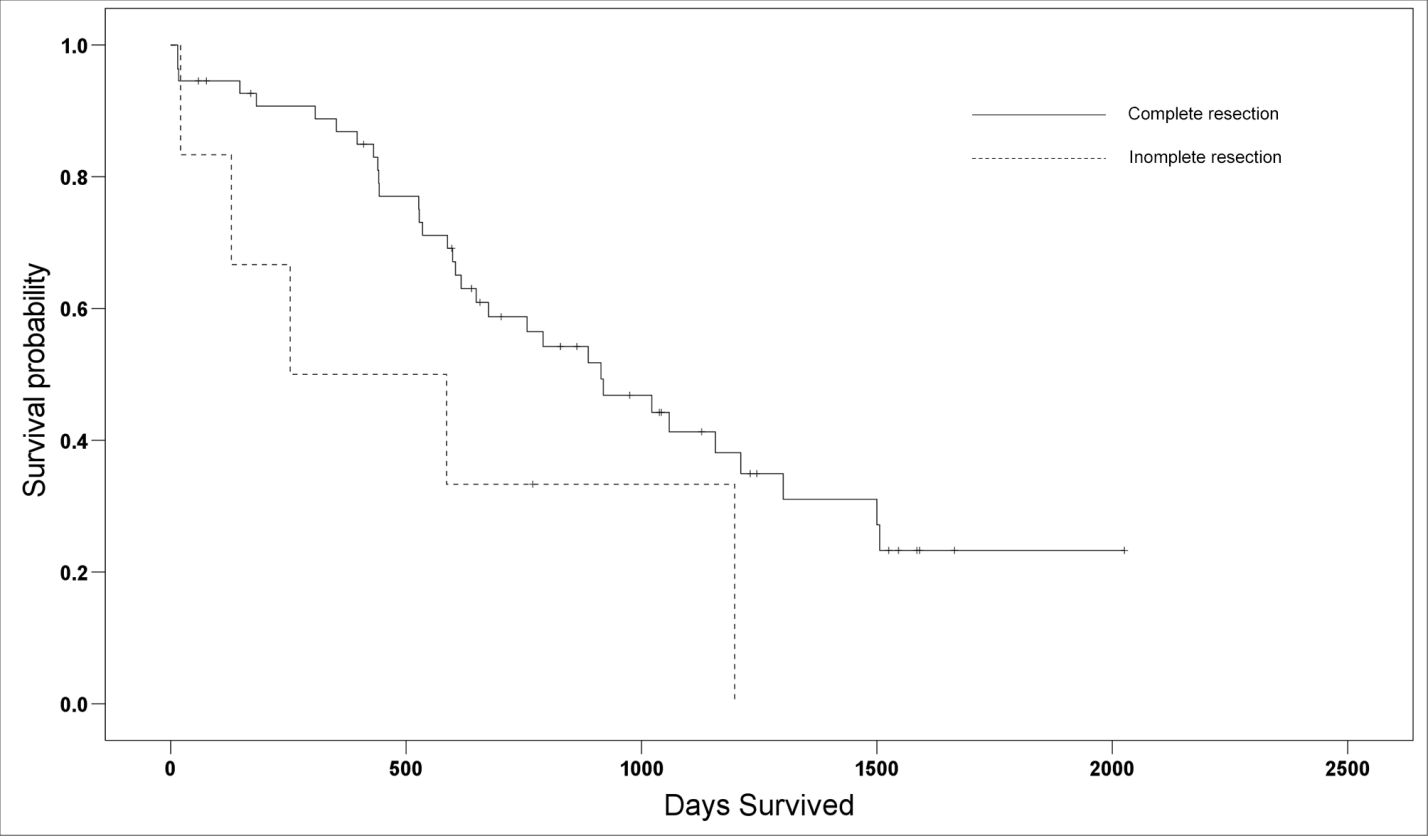
Kaplan–Meier survival curves for overall survival of dogs diagnosed with hepatocellular carcinoma that were surgically treated with complete (solid line) or incomplete (dashed line) resections. In cases of hepatocellular carcinoma, the median survival time among dogs with a complete resection (914 d) was significantly longer than that in those with an incomplete resection (254 d)

A follow-up was conducted for 104 dogs. The median follow-up was 598 d (range, 1–2,026 d). The presence or absence of recurrence was confirmed in 58 of the 104 dogs: 39 showed no recurrence, whereas 19 had recurrence. Early death during hospitalization occurred in 9 dogs. The causes of death in these cases included disseminated intravascular coagulation in 5 cases; hepatic dysfunction, renal failure, and aspiration pneumonia in 1 case each; and an unknown cause in 1 case. Of the 104 dogs, 61 had HCC, and their MST was 887 d (range, 15–2,026 d), excluding 6 cases with early death. Among the 61 dogs, the MST for 55 dogs with a complete resection was 914 d (range, 15–2,026 d), whereas that for 6 dogs with an incomplete resection was 254 d (range, 21–1,198 d) (Fig. 1). Thus, in cases of HCC, MST was significantly longer for dogs with a complete resection than those with an incomplete resection (*p* = 0.043).

## Discussion

In our study, fluorescence was frequently observed in cases of HCC, hepatocellular adenoma, hepatocholangiocarcinoma, and primary sarcoma (including undifferentiated sarcoma, hemangiosarcoma, and liposarcoma). In humans, poorly differentiated HCCs have been reported to show a ring-shaped fluorescence pattern [15]. This is thought to be due to the absence of tumor fluorescence caused by reduced ICG uptake. Conceivably, as in human HCC [15], in liver cells that had become cancerous, fluorescence was emitted because ICG excretion diminished while ICG uptake was preserved.

In a previous study involving the immunostaining of tissue with a ring fluorescence pattern in human HCC, ICG particles were observed in normal hepatic parenchyma surrounding the tumor [15]. However, in our study, canine HCCs showed both partial and total fluorescence patterns, and no differences in fluorescence patterns were observed due to differentiation. The differences between humans and dogs may be because human HCCs show decreased liver function, whereas canine HCCs generally do not. In the nodules that presented with a ring fluorescence pattern in our study (one case each of choledochal cyst, liver abscess, and primary sarcoma), mass-induced pressure on the surrounding normal liver tissue may have resulted in cholestasis, resulting in the accumulation of ICG particles in the surrounding area and, consequently, a ring fluorescence pattern. Furthermore, primary sarcomas and neuroendocrine tumors exhibited fluorescence; however, metastases and abscesses did not, and the frequency of fluorescence was also low in biliary tract masses. Human colorectal liver metastases have been reported to demonstrate a ring fluorescence pattern [15]. Regarding metastatic neoplasia in dogs, although the mass itself does not exhibit fluorescence, compression of the surrounding normal liver tissue may cause the mass to show a ring fluorescence pattern. The fluorescence that was observed frequently in nodular hyperplasia may have resulted from inhibited biliary excretion in hyperplastic tissue. Although a study using Chinese hamster ovary cells reported that organic anion transporting polypeptide 1B3 and sodium taurocholate cotransporting polypeptide were involved in the transport of ICG [30], this finding has not been demonstrated in canine cells. Determining the differences in fluorescence among hepatic masses in dogs may require in vitro studies using fluorescence microscopy.

The dose and timing of ICG administration to human liver tumors are still debatable. The most common dose is 0.5 mg/kg, but the timing of administration varies from 1 to 14 d before surgery [16]. In humans, administration within 24 h before surgery also increases the likelihood of false-positive results [16]; therefore, it is necessary to consider modifying the dosage and timing of administration in dogs.

Regarding the 10 cases in which the resection margin demonstrated fluorescence despite a complete resection, the margin surface may have fluoresced due to inhibited ICG excretion associated with the compression of normal liver cells. One case of hepatocholangiocarcinoma demonstrated bile duct expansion and mucus accumulation in the liver tissue surrounding the mass, which may have caused fluorescence. However, in all cases that did not demonstrate fluorescence of the resection margin, complete resection was achieved, suggesting that ICG fluorescence imaging may be effective for assessing complete resection in canine hepatic masses. Although histopathological diagnosis of resection margins is generally performed on sections obtained from the marginal tissues, ICG fluorescence may have potential for evaluating incomplete resection with macroscopical fluorescence findings of the resection margins. The dog liver is more segmented than the human liver. Therefore, it is possible to perform hepatic segment resection and hepatic lobectomy at the hepatic hilar. However, it may be left undetected if the tumor is close to the hilar. In such cases, ICG fluorescence can be used intraoperatively to detect this, and, if possible, an additional resection can be performed.

Some studies have reported an association between complete histopathological resection and surgical outcomes. Although two studies concluded that complete resection is not associated with surgical outcomes [22, 23], another study found that complete resection extended the MST [24]. In the present study, complete resection significantly extended the MST in the 61 dogs with HCC. This result suggests that complete resection is desirable when performing surgical resection of HCC in dogs, and that ICG fluorescence imaging in dogs with hepatic masses is effective for assessing complete resection and may be associated with treatment outcomes.

The limitations of this study include that the fluorescence of the resection margins could not be evaluated in all cases. In addition, there were a small number of cases with incomplete resection. Therefore, this study could not clarify the relationship between fluorescence and tumor invasion of the resection margins in canine HCC. This study also did not demonstrate the impact of ICG fluorescence imaging on the prognosis. After discharge from our hospital, the follow-up examinations depended on the owners. The recurrence and metastasis of HCC could not be accurately assessed in all cases; therefore, the relationship between ICG fluorescence of the resection margins and recurrence of HCC is still unclear. In addition, the causes of postoperative death after discharge were unidentified in some cases. Further investigations are warranted to clarify the clinical significance of ICG fluorescence imaging to the prognosis of canine patients with hepatic tumors.

## Conclusion

ICG fluorescence imaging has potential clinical value for the identification and margin evaluation of canine hepatic masses. Although fluorescence imaging was not available for differential diagnosis, it may be a promising tool for intraoperatively assessing complete resection in cases of hepatic masses demonstrating increased fluorescence in dogs, and complete resection of HCC could have a survival benefit. Further studies are necessary to determine the mechanism of ICG uptake in canine hepatic masses.

## Methods

### Patients

The patients comprised 104 privately owned dogs that were referred to Nihon University Animal Medical Center between October 2014 and October 2020 and diagnosed with hepatic masses for which surgical resection was indicated. Physical examination, hematology, serum chemistry, radiography, ultrasonography and computed tomography were performed in all dogs. After those examinations, all dogs underwent surgical removal of hepatic tumors. Data regarding signalment, clinicopathological findings, history of present illness, and past medical history were collected from medical records. Intraoperative ICG fluorescence imaging was performed before and after the removal of the hepatic mass.

Tissue samples were obtained from all the dogs for histopathological diagnosis. Prognosis of the dogs was confirmed by examining medical records or sending questionnaires to referring veterinarians and dog owners. Survival was defined as the number of days from surgery until death or termination of the study.

#### Informed consent

was obtained from all the owners and information about all the procedures were provided to the owners. All the procedures were approved by the Ethical Committee of Nihon University Animal Medical Center (accession No. ANMEC-3-011).

### Indocyanine green fluorescence imaging

**Fig. 2:**
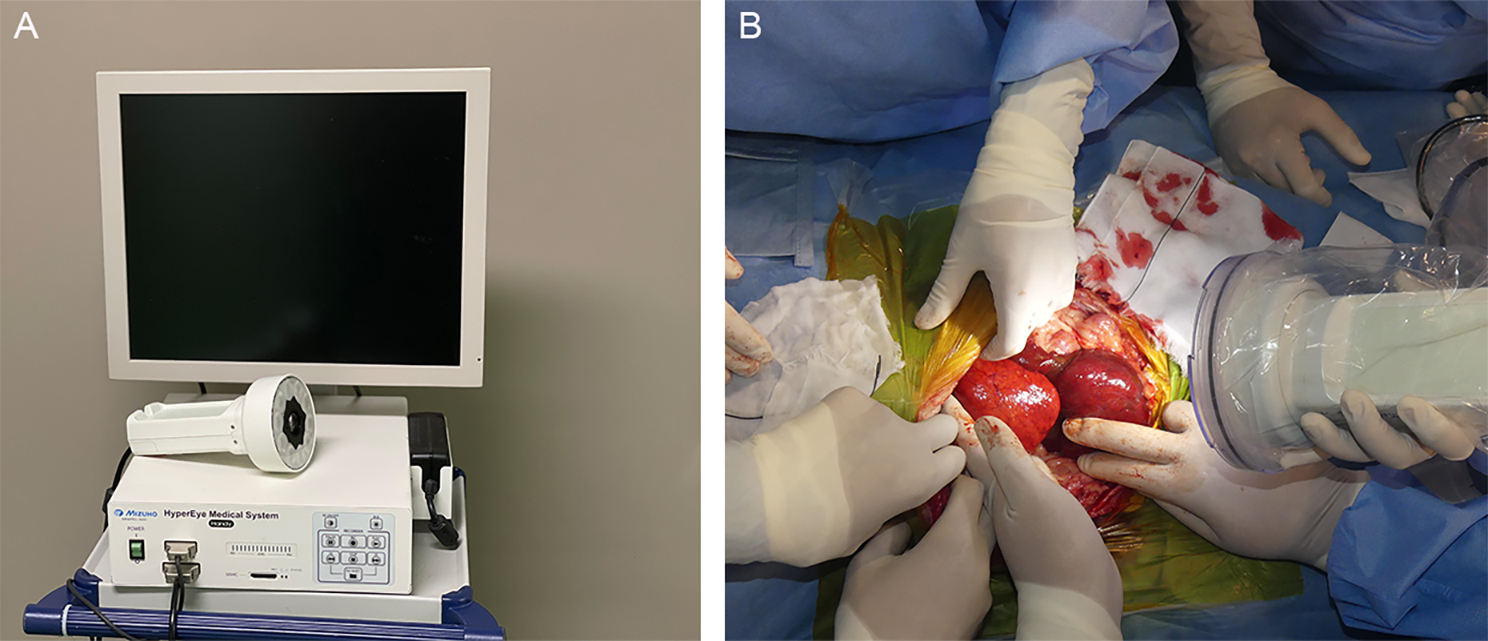
Near-infrared (NIR) fluorescence imaging using the HyperEye Medical System (HEMS). (A) Real-time NIR fluorescent images of the liver surface observed on the HEMS monitor. (B) Intraoperative observation of a hepatic fluorescent lesion. The camera unit was equipped with a light-emitting diode (LED) and charge-coupled device (CCD)

**Fig. 3:**
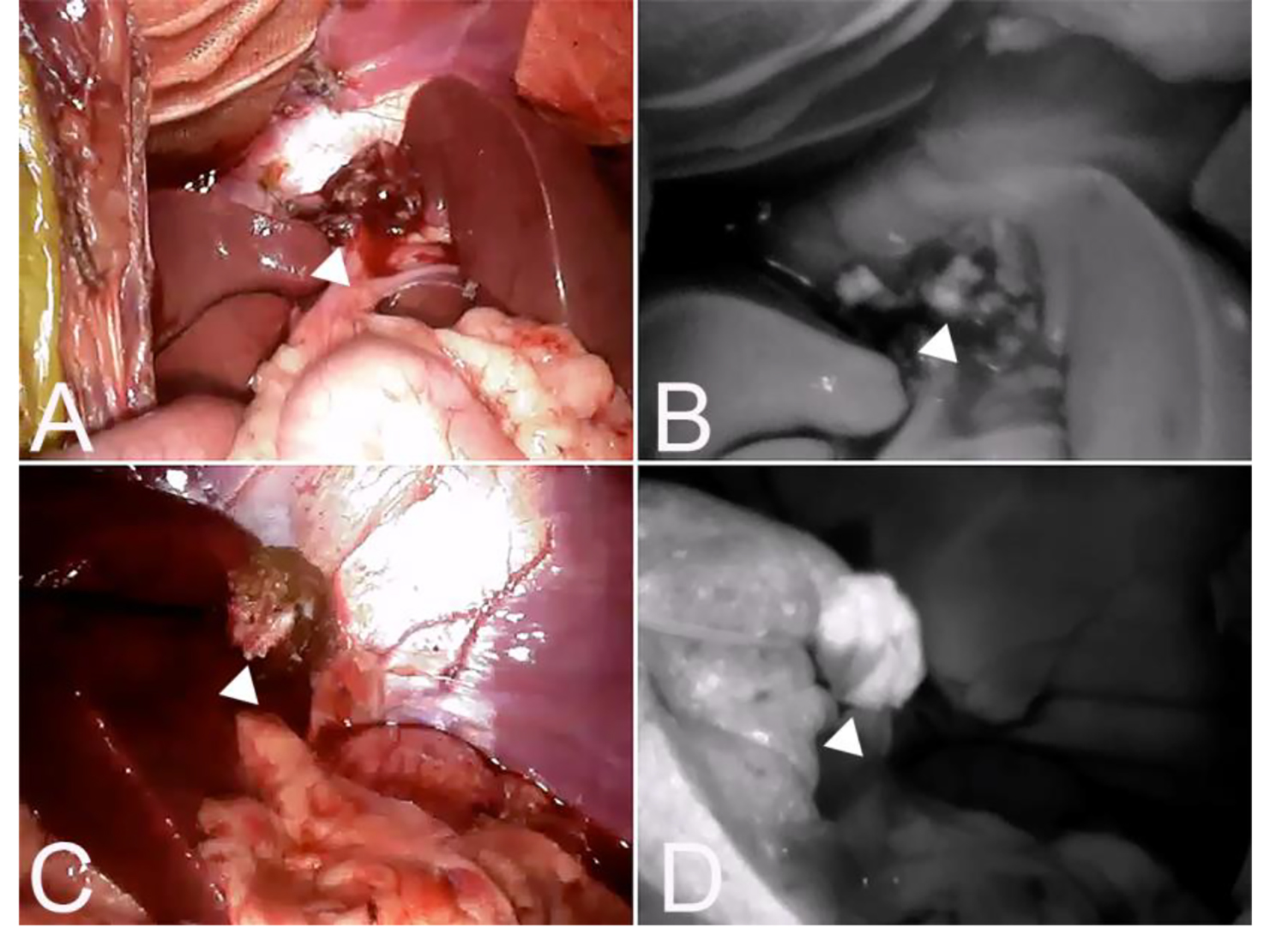
Fluorescence of the excised margin after excision of the tumor. A and C are the resection margins of hepatocellular carcinoma, and B and D are the fluorescence images of the resection margins of the same case. A (B) was a complete resection and no fluorescence was observed, whereas C (D) was an incomplete resection where fluorescence was observed

Between 12 − 24 h prior to surgery, ICG (Diagnogreen, Daiichi Sankyo Co. Ltd., Tokyo, Japan) was diluted to 5 mg/ml with water and administered intravenously at a dose of 0.5 mg/kg. The dose and timing of ICG administration were applied in accordance with a previous study on dogs with liver tumors [8]. During surgery, hepatic masses in the exposed livers were macroscopically observed after a Mercedes incision (cranial midline celiotomy and bilateral paracostal incisions). Before surgical resection of the hepatic masses, the surfaces of the livers were imaged using an infrared camera system (HyperEye Medical System; Mizuho Medical Co. Ltd, Tokyo, Japan) (Fig. 2). Images were captured with the surgical light turned off, and the camera was placed 30–50 cm from the surface of the liver. Hepatic masses were removed in all cases where surgical resection was indicated, regardless of fluorescence findings. After the hepatic masses were resected, the resection margins were imaged in the same manner (Fig. 3). The removed liver mass was placed in a shaded box, and the surface fluorescence was observed under the same conditions intraoperatively.

**Fig. 4:**
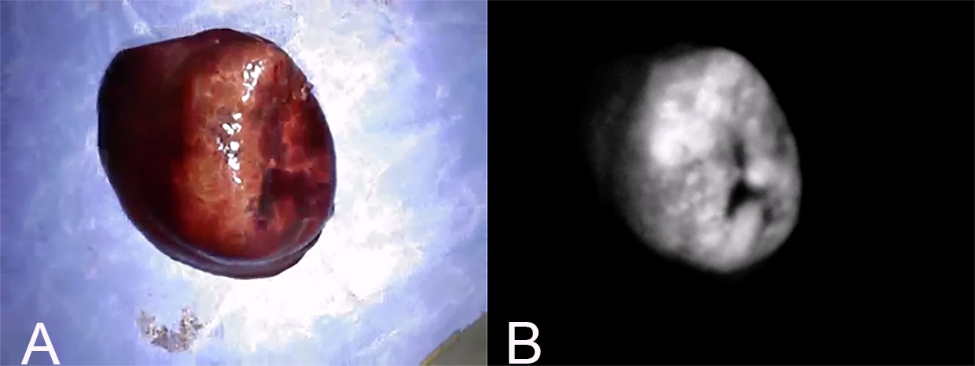
Fluorescence intensity 1. (A) Gross findings of a hepatic mass with fluorescence intensity 1. (B) Fluorescence imaging of the same mass. This pattern is defined when the fluorescence at the surface of the hepatic mass is increased in comparison with surrounding normal liver tissue

**Fig. 5:**
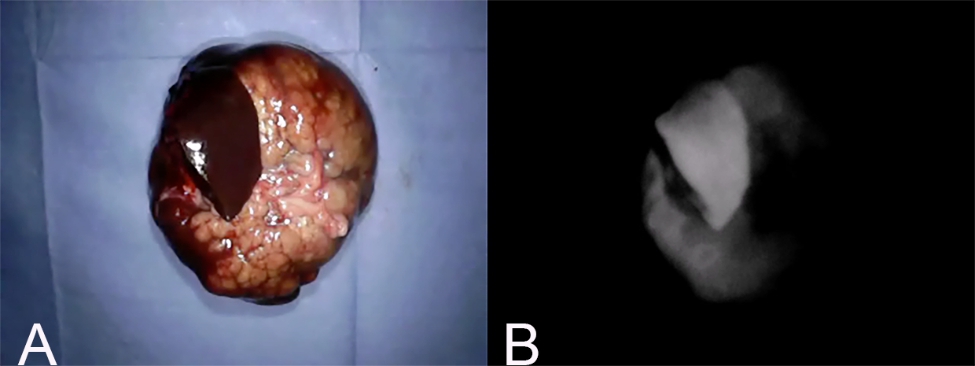
Fluorescence intensity 0. (A) Gross findings of a hepatic mass with fluorescence intensity 0. (B) Fluorescence imaging of the same mass. This pattern is defined when the hepatic mass surface fluorescence is equally intense in comparison with surrounding normal liver tissue

**Fig. 6:**
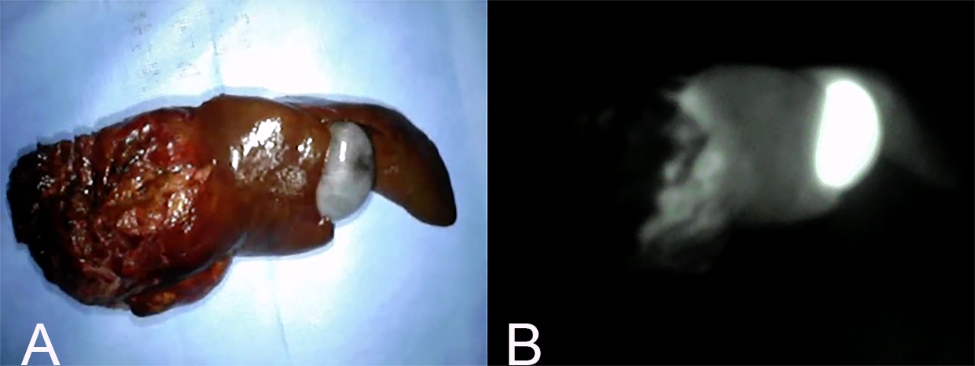
Fluorescence intensity − 1. (A) Gross findings of a hepatic mass with fluorescence intensity − 1. (B) Fluorescence imaging of the same mass. This pattern is defined when the fluorescence at the surface of the hepatic mass is decreased in comparison with surrounding normal liver tissues

Fluorescence intensity was defined as 1 if the fluorescence at the surface of the mass was increased compared to that of the surrounding normal liver tissue (Fig. 4); 0 if the fluorescence was equally intense (Fig. 5); and − 1 if the hepatic mass surface was decreased relative to the surrounding tissue (Fig. 6). A fluorescence intensity of 1 was defined as “fluorescence”, whereas 0 and − 1 were defined as “no fluorescence”.

**Fig. 7:**
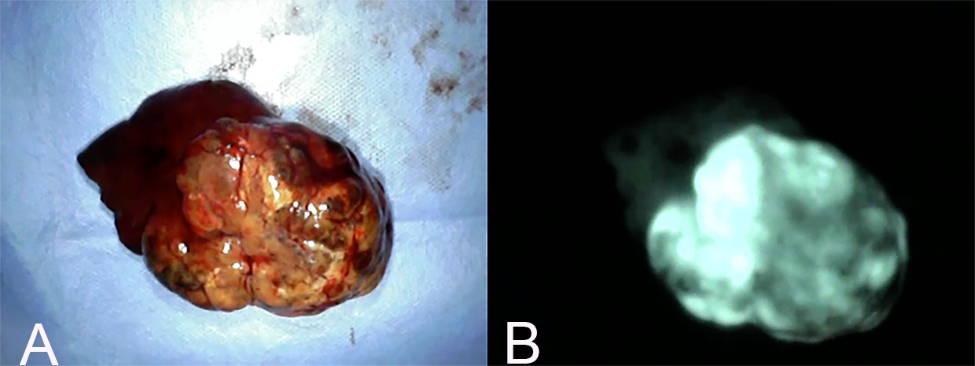
Partial fluorescence pattern. (A) Gross findings of a hepatic mass with partial fluorescence pattern. (B) Fluorescence imaging of the same mass. This pattern is defined when fluorescence is observed in mosaic form

**Fig. 8:**
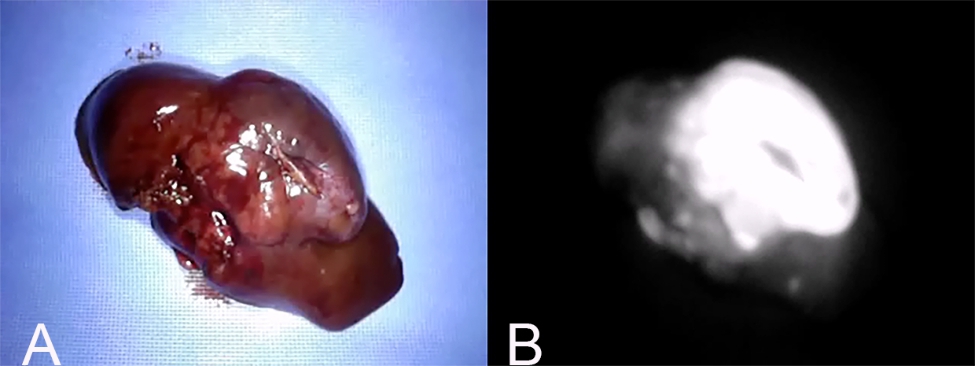
Whole fluorescence pattern. (A) Gross findings of a hepatic mass with whole fluorescence pattern. (B) Fluorescence imaging of the same mass. This pattern is defined when fluorescence is observed evenly throughout the image

**Fig. 9:**
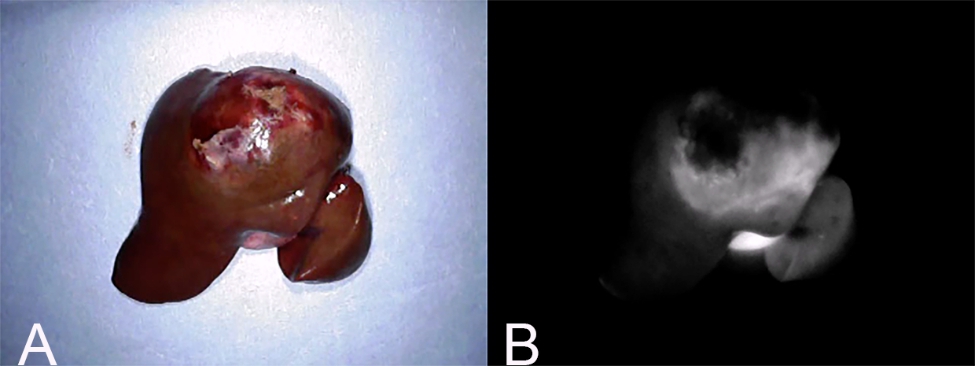
Ring fluorescence pattern. (A) Gross findings of a hepatic mass with ring fluorescence pattern. (B) Fluorescence imaging of the same mass. This pattern is defined when fluorescence is observed in the surrounding tissue but not in the center of the image

Fluorescence patterns were classified as “partial” if fluorescence was observed in mosaic form (Fig. 7); “whole” if fluorescence was observed evenly throughout the image (Fig. 8); and “ring” if fluorescence was observed in the surrounding tissue, but the center of the image lacked fluorescence (Fig. 9).

### Histopathological diagnosis

Tissue samples were immersed in 10% neutral buffered formalin for 48 h and then embedded in paraffin. After the sections were deparaffinized with xylene, they were immersed in ethanol. The slides were stained with hematoxylin and eosin and subjected to histopathological testing. Histopathological diagnosis was made by two boarded pathologists (YK and KO). Histopathological findings were then compared with fluorescence findings.

### Statistical analysis

Treatment outcomes were compared between cases of histopathologically complete resection and those without complete resection using the Kaplan-Meier method. Early death was defined as that within 2 wks of surgery. For surviving dogs, the point in time when the survival analysis was conducted was used as the censoring date. Early death cases were excluded from the survival analysis. The sensitivity and specificity of complete resection according to ICG fluorescence imaging were calculated for nodules for which the fluorescence of the resection margin was assessed. Statistical analyses were performed using SPSS Statistics (IBM, Brussels, Belgium). Statistical significance was set at *p* < 0.05.

## Declarations

### Data availability

All data supporting the conclusions of this article are included within the article.

## List of abbreviations

HCC: hepatocellular carcinoma
ICG: indocyanine green
MST: median survival time
